# Extreme Heat and COVID-19: A Dual Burden for Farmworkers

**DOI:** 10.3389/fpubh.2022.884152

**Published:** 2022-05-04

**Authors:** David López-Carr, Jennifer Vanos, Armando Sánchez-Vargas, Río Vargas, Federico Castillo

**Affiliations:** ^1^Department of Geography, University of California, Santa Barbara, Santa Barbara, CA, United States; ^2^School of Sustainability, Arizona State University, Tempe, AZ, United States; ^3^Institute of Economic Research, National Autonomous University of Mexico, Mexico City, Mexico; ^4^University of California, Berkeley, Berkeley, CA, United States

**Keywords:** COVID-19, extreme heat, farm labor, dual burden of disease, farm work, EPI-epidemiology, public health

## Abstract

Currently, there is an extensive literature examining heat impacts on labor productivity and health, as well as a recent surge in research around COVID-19. However, to our knowledge, no research to date examines the dual burden of COVID-19 and extreme heat on labor productivity and laborers' health and livelihoods. To close this research gap and shed light on a critical health and livelihood issue affecting a vulnerable population, we urge researchers to study the two topics in tandem. Because farmworkers have a high incidence of COVID-19 infections and a low rate of inoculation, they will be among those who suffer most from this dual burden. In this article, we discuss impacts from extreme heat and COVID-19 on farm laborers. We provide examples from the literature and a conceptual framework showing the bi-directional nature of heat impacts on COVID-19 and vice versa. We conclude with questions for further research and with specific policy recommendations to alleviate this dual burden. If implemented, these policies would enhance the wellbeing of farmworkers through improved unemployment benefits, updated regulations, and consistent implementation of outdoor labor regulations. Additionally, policies for farmworker-related health needs and cultural aspects of policy implementation and farmworker outreach are needed. These and related policies could potentially reduce the dual burden of COVID-19 and extreme heat impacts while future research explores their relative cost-effectiveness.

## Introduction

There are a host of studies examining heat impacts on labor productivity and health [e.g., see (1)], as well as a recent spate of research around COVID-19. In the case of farmworkers in California's Central Valley Region, results show that an increase of the heat index from 95°F to 100°F can result in losses of agricultural productivity between 4 and 8%, depending on labor intensity of the crop (2). For COVID-19, the impact of agricultural workers' reduction of hours of labor has been estimated to be ~$301 M as of March, 2021 (2). Furthermore, pre-existing conditions that make farmworkers vulnerable to heat also make them vulnerable to the impacts of COVID-19. Among them are obesity, smoking status, cardiovascular conditions, and diabetes. While temperature and humidity may be inversely related to COVID-19 transmission (3–5), it remains unclear if this is due to changes in human behavior (e.g., more people clustering indoors during colder periods).

However, to our knowledge, no research has examined the dual burden of COVID-19 and extreme heat on labor productivity and laborers' health and livelihoods. And yet, relative to the rest of the country, COVID-19 infections are among the highest and inoculation rates among the lowest among migrant agricultural workers in the United States (6). This, is a quintessential example of environmental injustice with farmworkers—not consumers or land owners—among those who most suffer from this dual burden. The scholarly community has an opportunity to reconcile this deficiency with more research examining the coupling of the two health burdens.

With anomalous heat events due to climate change on the increase, farmworkers may lose more days of work in the future. COVID-19 will increase the number of days lost and may also worsen physical symptoms during high heat periods. With extreme heat events, the body suffers water loss and potential kidney damage (7). Adding COVID-19 to extreme heat, in the short term, could exacerbate morbidity and increase mortality by stressing the body beyond its capacity to deal with either burden adequately, much less the combination of the two. Does the twin burden of having suffered both simultaneously worsen the symptoms of one, the other, or both? Moreover, in the long term, does organ damage from excessive heat exposure—where heatstroke can damage heart, kidneys, liver, and the brain (8)—intensify symptoms of “long” COVID, symptoms of which we are not yet aware? To our knowledge, no research presently exists to answer these questions.

## What We Know About COVID-19 and Migrant Farmworkers

COVID-19 has a detrimental effect on our most vulnerable communities, including essential agricultural laborers, considered essential workers in the U.S. (9). Due to the nature of their job, agricultural workers tend to have numerous points of physical contact and few opportunities for social distancing in the workplace. Moreover, living conditions for the great majority of farmworkers are substandard and often temporary as they move from place-to-place following crop harvesting (10). Farmworkers have experienced a comparably higher rate of COVID-19 infections relative to the general population. For example, in Monterrey County, California the observed rate of positive test results for Latino/a farm workers was 22% while that of the rest of the county population was 6% in November-December 2020 (11, 12). Additionally, it is not possible to carry out this form of agricultural labor remotely or virtually, making farmworkers more susceptible to infections in the workplace. The negative impact of COVID-19 on farmworkers is exacerbated by a decrease in the availability of farmworkers and an increase in their average age (13).

Vaccine hesitancy contributes to the negative impacts of COVID-19 on the workforce of California's food supply. In Monterrey County, for example, only about 50% of farmworkers stated they were very likely to get vaccinated, while studies show that the same figure for the rest of the country is 69% (12). In more rural areas of the county, women and younger farmworkers are more vaccine-hesitant, largely due to a distrust of the government, lack of information on the effectiveness of the vaccine, and concerns with side effects (12).

The pandemic poses significant economic impacts to migrant farmworkers and their families. For example, when farmworkers become ill due to COVID-19, lose work hours, productivity, and income. Workers often need to continue working even when testing positive for COVID-19 (12). Working while infected with COVID-19 is likely to result in lower worker productivity, resulting in income loss (14). This action also presents an additional risk of infection to the rest of the agricultural workers, which multiplies the financial impact, as well as the long-term health impacts of COVID-19 on the agricultural labor force. Furthermore, farmworker income reduction due to COVID-19 restrictions and infection impacts not only farmworkers but also those who depend on the farmworker financially. For example, an impacted farmworker may become unable to pay for basic needs such as food, housing, electricity, and/or water for the farmworker's family and/or other household members. Furthermore, the negative impacts of COVID-19 expand beyond the immediate family of the farmworker to those who depend on remittances in their home countries.

## What We Know About Heat and Migrant Farmworkers

Migrant farmworkers, particularly in the Central Valley of California and southern U.S. states, endure extreme heat during the summer and early fall. Prolonged exposure to intense heat combined with physical labor can be deadly to agricultural workers, and numerous migrant worker deaths occur each year due to these circumstances. The CDC reports that heat deaths among crop workers are 20 times greater than among U.S. civilian workers, even when farms are in compliance with heat-related safety regulations (15). Moreover, research has shown that from 2005–2014, 198 non-U.S. citizen deaths occurred on farms, which is over triple that of U.S. citizens (16). Further, many of these deaths and illnesses may not be counted due to cause of death (heat-caused vs. heat-related), linguistic or cultural barriers, immigration status, and other factors (1).

Exposure to heat over time can lead to severe illnesses such as heatstroke, dehydration, cardiovascular disease, and critical organ failure (17). Moreover, farmworkers may be more susceptible to extreme heat illness due to risk factors such as low-income, male gender, migration status, type of work at the farm, and pre-existing illness, including potentially related cardiovascular disease, kidney disease, and diabetes (18, 19). These negative impacts of heat on agricultural workers are significant and continue to increase due to a warming climate and growing demand for agricultural labor in fertile agricultural areas of the U.S., particularly during the harvesting months, which coincide with the most extreme conditions. Research shows that the physical work capacity of an agricultural laborer lowers as the air temperature, humidity, and sunlight increase (20, 21). Thus, as more agricultural laborers are hired to compensate for lost work productivity, a greater number of vulnerable outdoor workers are negatively impacted by heat and require essential coping resources (2).

## How Might COVID-19 Exacerbate Heat-Health Issues?

**Figure 1:**
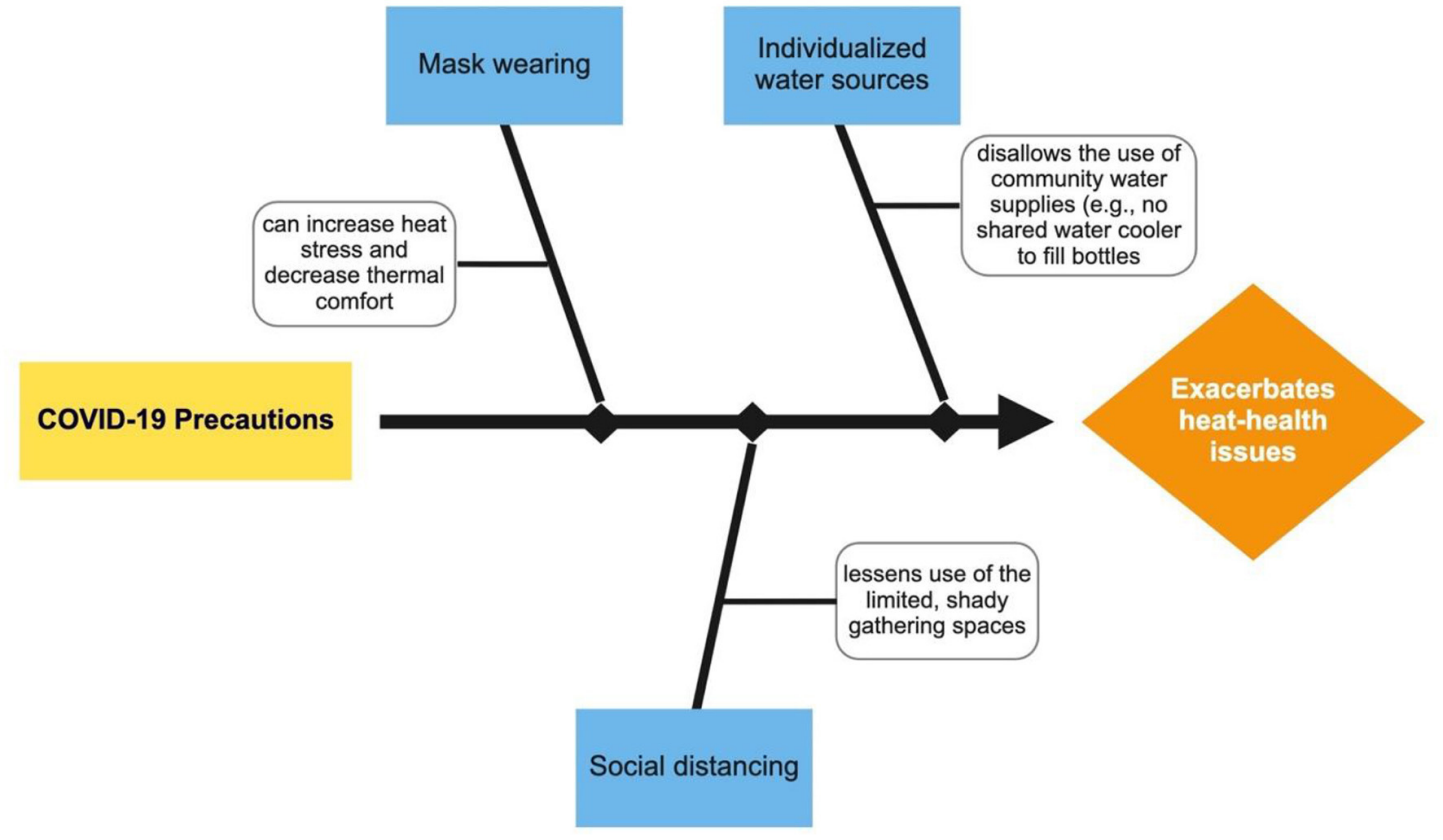
How COVID-19 precautions can exacerbate heat-health issues.

Figure 1 presents a conceptual framework of the pathways by which COVID-19 can exacerbate heat-health Issues. These essential workers are already more vulnerable than the general population to the effects of heat due to potential pre-existing illness, lack of health care, and lack of coping resources (10, 11). COVID-19 precautions may exacerbate the vulnerabilities due to less use of cooling resources (e.g., cooled or shady spaces that require individuals to gather) and may disallow the use of community water supplies (e.g., no shared cooler to fill bottles). Discomfort in the heat from mask wearing is well-known and can increase heat stress and thermal discomfort while potentially limiting water intake during breaks.

## Does Extreme Heat Make Getting COVID-19 More Likely?

**Figure 2:**
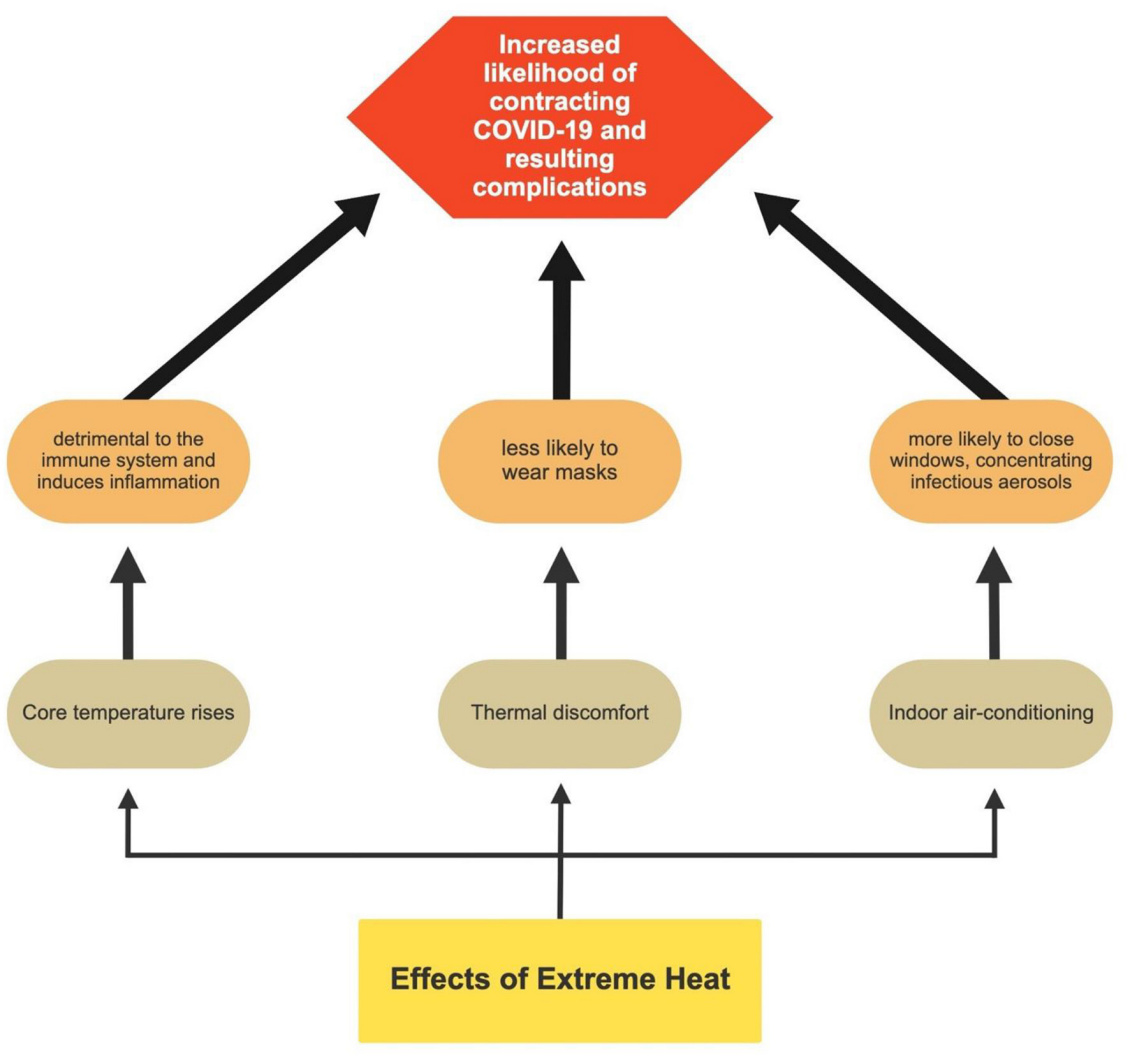
Pathways through which extreme heat can increase the probability of contracting COVID-19.

Extreme heat may compound COVID-19 infection rates for several reasons, which are conceptually outlined in Figure 2. First, overheating of the body corresponding to core temperatures >38°C is commonly found in outdoor workers (22, 23). Increased core temperatures can have detrimental effects on the immune system's performance, which can be further compromised when heat stress is combined with exertion. Overheating induces inflammation and prevents inflammatory resolution (24). Hence, a lowered immune system increases the likelihood of contracting COVID-19 and may increase resulting complications.

Second, during periods of high temperatures, when not working in the fields, agricultural workers are more likely to use indoor air conditioning, if available. This action prevents the flow of outside air and concentrates infectious aerosols (25). Third, under higher heat, farmworkers may be less likely to wear masks due to discomfort. Decreased mask wearing increases the potential for infection. Those who are infected breathe out infectious aerosols into the free air and vulnerable, unmasked individuals breathe in those particles directly. Lastly, in warm weather, aerosols and water droplets may remain airborne longer; conversely, in colder weather when the air is heavier, aerosols may be denser and sink downward (25, 26).

**Figure 3:**
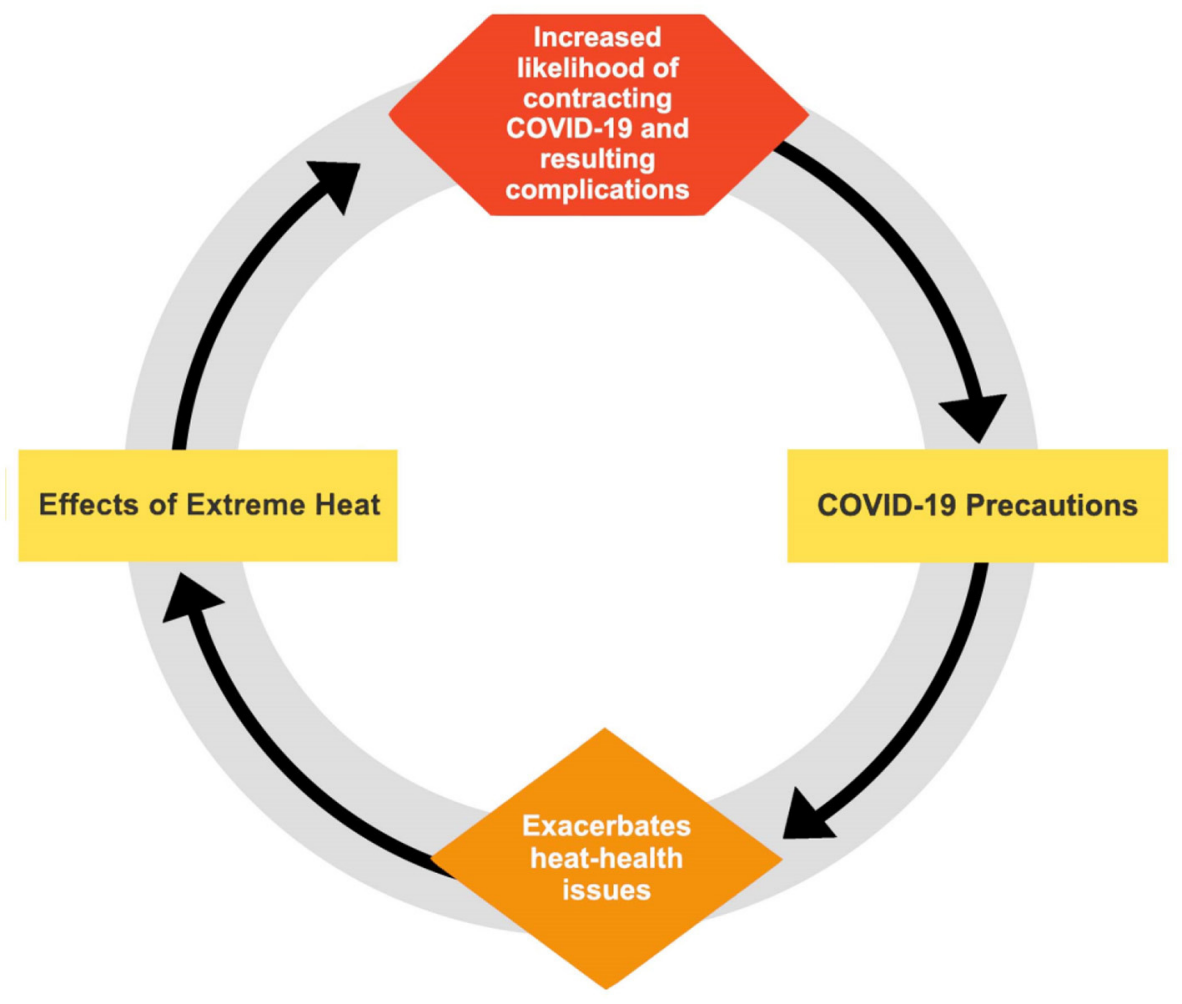
Joint Effects of heat and COVID-19 on human health.

As observed in Figure 3, we consider the relation between heat and COVID-19 to be bi-directional. As described above, contracting COVID-19 may exacerbate heat-related health challenges and, conversely, excessive heat exposure may exacerbate contracting COVID-19. To the extent the heat exposure is extreme, the impacts of contracting COVID-19 may also increase. Similarly, when a COVID-19 outbreak is large or an individual is shedding a large viral load, the deleterious impacts on heat exposure can be increased. More research is needed to determine the relative strength of these linkages.

## Discussion

Despite a burgeoning literature on COVID-19, more research is needed to address the burden of COVID-19 in concert with anomalous heat, labor productivity, and laborers' health and livelihoods. We discussed potential pathways of how heat can impact COVID-19 morbidity and mortality and vice versa. We argue that this twin burden further impacts labor productivity and farmworker livelihoods in an insidious cycle, exceeding the sum of each illness independently.

More research integrating the two topics is warranted. Many largely unanswered questions remain to be examined by researchers. For example, under what circumstances does COVID-19 lead to increased heat health challenges? Specifically, to what extent does mask-wearing, social distancing, and individualized (or not) water consumption lead to COVID-19 heat stress? To what degree may there be a connection between long-haul COVID and heat susceptibility and associated consequences in labor productivity? What other factors may impact heat health challenges? More research is needed also to address the impacts of heat stress on COVID-19 morbidity and mortality. For example, to what extent does heat stress induced immunodeficiency facilitate COVID-19 infection? To what extent is heat stress likely to reduce mask wearing and therefore increase COVID-19 infection rates? To what degree does extreme heat lead to increased use of air-conditioning, subsequent concentration of aerosols in an enclosed space and ultimately, to potentially increased COVID-19 prevalence? What other heat-related health factors might exacerbate COVID-19 morbidity and mortality? The dearth of data on these topics calls for new empirical field research combining surveys and interviews with climatological data.

Beyond the knowledge gap and opportunity for researchers on the topic, direct policy implications result from this dual burden. The similarity of socio-economic and labor impacts of COVID-19 and heat stress, allows policymakers to design and implement policies for both with a broader impact on farmworkers' wellbeing. For example, unemployment benefits, if implemented for farmworkers, can have a broader positive impact on the target population as they recover from either or both health conditions. Consideration of the expansion of worksite policies such as expanding paid sick leave for impacted workers could also be useful. Lessons learned from implementing policies related to heat stress (e.g., cancellation or change of activities, heat mitigation, personal cooling, other coping/adaptation strategies, etc.) may provide guidance for policies related to COVID-19. Therefore, public health policies and workplace, acclimatization protocols can be informed in ways that ameliorate suffering from both COVID-19 and cognate infectious diseases as well as heat stress. These strategies must be place-based to assess and lessen both burdens as they intersect with outdoor labor conditions, farmworker related health needs, and cultural aspects of policy implementation and farmworker outreach. Intervention points or policy levers can be leveraged suitably. For example, the timing of worksite vaccination campaigns can be planned to coincide with workplace heat prevention acclimatization protocols, raising awareness among workers and employers about the dangers of both stressors. Understanding the intersection of COVID-19 and climate change via heat stress results in an opportunity for policy makers to design and implement policies that may have greater impact when addressing the health and socio-economic impacts of both occurrences.

## Data Availability Statement

The original contributions presented in the study are included in the article/supplementary material, further inquiries can be directed to the corresponding author/s.

## Author Contributions

DL-C drafted the conceptualization of the article. DL-C, JV, AS-V, and FC wrote the article. All authors contributed to the article and approved the submitted version.

## Funding

This work was supported by University of California, Research Programs on Migration and Health (PIMSA) Award University of California Institute for Mexico and the United States (UC MEXUS)-CONACYT Collaborative Grant Binational Collaborative Projects Addressing COVID-19. The National Autonomous University of Mexico (UNAM) through the Scientific Research Coordination (CIC), the Liaison and Technology Transfer Coordination (CVTT) and the University of California (UC) through Alianza UCMX in collaboration with the Secretariat of Foreign Affairs of Mexico Convocatoria Extraordinaria de Colaboración Binacional frente al COVID-19, UNAM—Universidad de California (UC).

## Conflict of Interest

The authors declare that the research was conducted in the absence of any commercial or financial relationships that could be construed as a potential conflict of interest.

## Publisher's Note

All claims expressed in this article are solely those of the authors and do not necessarily represent those of their affiliated organizations, or those of the publisher, the editors and the reviewers. Any product that may be evaluated in this article, or claim that may be made by its manufacturer, is not guaranteed or endorsed by the publisher.

## References

[1] Vanos J Moyce S Lemke B Kjellstrom T. Extreme Heat Exposure and Occupational Health in a Changing Climate. In: Castillo F, Wehner MF and Stone D. Extreme Events and Climate Change: A Multidisciplinary Approach. Washington, D.C.: John Wiley and Sons (2021). p. 147–66.

[2] Castillo F Sanchez A Gilless JK Wehner MF. The Impact of Heat Waves on Labor Productivity and Output. Castillo F, Wehner MF and Stone D, Extreme Events and Climate Change: A Multidisciplinary Approach. Washington, D.C.: John Wiley and Sons (2021). p. 11–20.

[3] Notari A. Temperature dependence of COVID-19 transmission. Sci Total Environ. (2021) 763:144390. 10.1016/j.scitotenv.2020.144390 33373782 PMC7733690

[4] Tobías A Molina T. Is temperature reducing the transmission of COVID-19?. Environ Res. (2020) 186:109553. 10.1016/j.envres.2020.109553 32330766 PMC7165096

[5] Liu J Zhou J Yao J Zhang X Li L Xu X. Impact of meteorological factors on the COVID-19 transmission: A multi-city study in China. Sci Total Environ. (2020) 726:138513. 10.1016/j.scitotenv.2020.138513 32304942 PMC7194892

[6] Lusk JL Chandra R. Farmer and farm worker illnesses and deaths from Covid-19 and impacts on agricultural output. PLoS ONE. (2021) 16:e0250621. 10.1371/journal.pone.0250621 33909685 PMC8081247

[7] Glaser J Lemery J Rajagopalan B Diaz HF Garcia-Trabanino R Taduri G. Climate change and the emergent epidemic Ckd from heat stress in rural communities: the case for heat stress nephropathy. Clin J Am Soc Nephrol. (2016) 11:1472–83. 10.2215/CJN.13841215 27151892 PMC4974898

[8] Bouchama A Knochel JP. Heat stroke. N Engl J Med. (2002) 346:1978–88. 10.1056/NEJMra011089 12075060

[9] Haley E Caxaj S George G Hennebry J Martell E McLaughlin J. Migrant farmworkers face heightened vulnerabilities during COVID-19. JAFSCD. (2020) 9:35–9.

[10] Castillo F Mora AM Kayser GL Vanos J Hyland C Yang AR. Environmental Health Threats to Latino Migrant Farmworkers. Annu Rev Public Health. (2021) 42:257–76. 10.1146/annurev-publhealth-012420-105014 33395542 PMC8168948

[11] Mora AM Lewnard JA Kogut K Rauch S Morga N Hernandez S. Risk factors for Sars-Cov-2 infection among farmworkers in monterey county, California. medRxiv. (2021). 10.1101/2021.02.01.21250963 34524438 PMC8444020

[12] Mora AM Lewnard JA Kogut K Rauch S Morga N Jewell N. Impact of the Covid-19 pandemic and vaccine hesitancy among farmworkers from monterey County, California. medRxiv. (2020). 10.1101/2020.12.18.20248518

[13] Martin P. Covid-19 and California Farm Labor. Calif Agric. (2020) 74:67–8. 10.3733/ca.2020a0017

[14] Pak A Adegboye OA Adekunle AI Rahman KM McBryde ES Eisen DP. Economic consequences of the Covid-19 outbreak: the need for epidemic preparedness. Front Public Health. (2020) 8:241. 10.3389/fpubh.2020.00241 32574307 PMC7273352

[15] Langer CE Mitchell DC Armitage TL Moyce SC Tancredi DJ Castro J. Are Cal/Osha regulations protecting farmworkers in california from heat-related illness? J Occup Environ Med. (2021) 63:532–39. 10.1097/JOM.0000000000002189 33741829 PMC8893044

[16] Taylor EV Vaidyanathan A Flanders WD Murphy M Spencer M Noe RS. Differences in heat-related mortality by citizenship status: United States, 2005–2014. Am J Public Health. (2018) 108:S131–S36. 10.2105/AJPH.2017.304006 29072944 PMC5920731

[17] Mac V Elon L Mix J Tovar-Aguilar A Flocks J Economos E. Risk factors for reaching core body temperature thresholds in Florida agricultural workers. J Occup Environ Med. (2021) 63:395–402. 33560064 10.1097/JOM.0000000000002150

[18] Kuehn BM. Why farmworkers need more than new laws for protection from heat-related illness. JAMA. (2021) 326:1135–7. 10.1001/jama.2021.15454 34495301

[19] Culp K Tonelli S Ramey SL Donham K Fuortes L. Preventing heat-related illness among hispanic farmworkers. AAOHN J. (2011) 59:23–32. 10.1177/216507991105900104 21229935

[20] Masuda YJ Garg T Anggraeni I Ebi K Krenz J Game ET. Warming from tropical deforestation reduces worker productivity in rural communities. Nat Commun. (2021) 12:1–8. 10.1038/s41467-021-21779-z 33707454 PMC7952402

[21] Zhu J Shuo W Zhang B Wang D. Adapting to changing labor productivity as a result of intensified heat stress in a changing climate. Geohealth. (2021) 5:5e2020GH000313. 10.1029/2020GH000313 33817537 PMC8011619

[22] Kjellstrom T Holmer I Lemke B. Workplace heat stress, health and productivity-an increasing challenge for low and middle-income countries during climate change. Global Health Action. (2009) 2:2047. 10.3402/gha.v2i0.2047 20052422 PMC2799237

[23] Vega-Arroyo AJ Mitchell DC Castro JR Armitage TL Tancredi DJ Bennett DH. Impacts of weather, work rate, hydration, and clothing in heat-related illness in California farmworkers. Am J Ind Med. (2019) 62:1038–46. 10.1002/ajim.22973 30964208

[24] Presbitero A Melnikov VR Krzhizhanovskaya VV Sloot P. A unifying model to estimate the effect of heat stress in the human innate immunity during physical activities. Sci Rep. (2021) 11:1–18. 10.1038/s41598-021-96191-0 34404876 PMC8371171

[25] Kohanski Lo MA Waring MS. Review of indoor aerosolo generation transport, and control in the context of Covid-19. Int Forum Allergy Rhinol. (2020) 10:1173–79. 10.1002/alr.22661 32652898 PMC7405119

[26] Dabisch P Schuit M Herzog A Beck K Stewart W Krause M. The Influence of Temperature, Humidity, and Simulated Sunlight on the Infectivity of Sars-Cov-2 in Aerosols. Aerosol Sci Technol. (2021) 55:142–53. 10.1080/02786826.2020.1829536 PMC10698725 38077296

